# Booze, Bars, and Bystander Behavior: People Who Consumed Alcohol Help Faster in the Presence of Others

**DOI:** 10.3389/fpsyg.2016.00128

**Published:** 2016-02-11

**Authors:** Marco van Bommel, Jan-Willem van Prooijen, Henk Elffers, Paul A. M. Van Lange

**Affiliations:** ^1^Department of Experimental and Applied Psychology, VU University AmsterdamAmsterdam, Netherlands; ^2^Netherlands Institute for the Study of Crime and Law EnforcementAmsterdam, Netherlands; ^3^Department Psychology of Conflict, Risk, and Safety, University of TwenteEnschede, Netherlands

**Keywords:** bystander effect, bystander intervention, alcohol, helping, disinhibition

## Abstract

People help each other less often and less quickly when bystanders are present. In this paper, we propose that alcohol consumption could attenuate or reverse this so-called bystander effect. Alcohol impairs people cognitively and perceptually, leading them to think less about the presence of others and behave less inhibited. Moreover, alcohol makes people more prone to see the benefits of helping and not the costs. To provide an initial test of these lines of reasoning, we invited visitors of bars in Amsterdam to join our study at a secluded spot at the bar. We manipulated bystander presence, and at the end of the study, we measured alcohol consumption. When participants took their seats, the experimenter dropped some items. We measured how many items were picked up and how quickly participants engaged in helping. Results revealed that alcohol did not influence the bystander effect in terms of the amount of help given. But importantly, it did influence the bystander effect in terms of response times: people who consumed alcohol actually came to aid *faster* in the presence of others.

## Introduction

Imagine having dinner with three colleagues in a nice, but crowded restaurant. Suddenly you hear some loud coughing sounds. The sounds seem to come from a man who sits alone at a table not too far from yours. Slowly his face turns red, and you suspect he is choking. Would you leave your colleagues and help this man? From what we know about the bystander effect, especially when other people are around, helping is unlikely in this situation, or it may take a long time before someone helps. In the present study, however, we examine the possibility that if the dinner was accompanied by some glasses of wine, people may in fact be quite ready to intervene, even when other bystanders are around.

Alcohol consumption is often seen as one of the most important determinants of anti-social behaviors such as vandalism and interpersonal violence (Bushman, 1997; Graham et al., 1998). Indeed, research on violent offending consistently shows that a large proportion of all violent acts take place in pubs and clubs where large quantities of alcohol may be consumed (Allen et al., 2003). Consuming alcohol, however, does not always induce antisocial behavior (Felson et al., 2011). In this paper, we discuss the possibility that the consumption of alcohol can have an opposite effect: alcohol consumption in some contexts could lead to pro-social behavior. More specifically, we will study the effects of alcohol consumption in the context of the bystander effect.

### The Bystander Effect

Since the early work of Darley and Latané (1968) it has become clear that the presence of bystanders exerts a negative influence on helping or other forms of intervention behavior: when there are more bystanders, people help less frequently. At the same time, research shows that people also make the decision to help less quickly when there are others around, even though a quick response could have completely changed the outcome and, for example, save a victim from a heart attack.

The bystander effect became publicly known through news reports about bystanders of violent crimes who neglected to intervene sufficiently or timely. For instance, Latané and Darley (1968) started their research into the bystander effect after a young nurse was violently raped and murdered (for details, see Manning et al., 2007). Because interpersonal violence often takes place at social gatherings where drinking is involved (Allen et al., 2003), to fully understand bystander behavior, we must understand the effects of alcohol on intervening in the presence of others. In this paper, we outline and empirically test the notion that alcohol consumption can cause bystanders to overcome the inhibitory effects of the presence of others in helping situations.

The decision to help or not is often based on an implicit calculation in which people outweigh the (emotional) costs and benefits of helping, versus those of not helping (Dovidio et al., 1991). Latané and Darley (1970) developed a multi-stage model which describes bystander intervention during an emergency on a step by step basis: (1) Noticing the emergency; (2) Interpreting it as an actual emergency; (3) Taking responsibility to help; (4) Deciding how to help; (5) Implementing the help. At each of the five steps, when others are present, several psychological processes make the balance between the perceived costs and benefits of helping versus not helping, tip toward the latter. We outline the three main processes.

First, a well-known process is referred to as *diffusion of responsibility.* People have a diminished sense of responsibility as they attribute the responsibility across other bystanders. Responsibility is shared, which clouds a feeling of personal responsibility. Moreover, sometimes people are confident that someone else will intervene, and thus there is no need for them to intervene themselves, but other times people ask themselves why they have to be the one to intervene when no-one else does (e.g., Weesie, 1993). For instance, in the dinner example we started this paper with, why would you go and ask if the man needs help while the waiter staff, or a colleague could also do it? Second, another process which makes it less likely that people take the next step is *pluralistic ignorance*. This entails that people look at the behavior of others to gage the correct or normative response in a situation. Because no-one in the restaurant is helping the man who may be choking, it could lead to everyone thinking that helping the man is inappropriate, thus causing people to intervene much too late or not at all (Latané and Rodin, 1969; Clark and Word, 1972). Third, there is *audience inhibition*, which entails that people feel inhibited when there is an audience (other bystanders) around, because they fear to be evaluated negatively when they decide to step out of the crowd and intervene.

Audience inhibition can happen for at least two reasons. First, people fear they misinterpret the situation and help is actually not required. The choking man in the restaurant could just be coughing and looking red because he has a fever. Indeed, in a study by Tice and Baumeister (1985), it was unclear for actual participants whether or not an ostensible fellow participant was choking and needed help. They found that, in particular, highly masculine people were less likely to help, because they may have been more concerned about losing poise, and become embarrassed if their help proved unnecessary. The second reason for audience inhibition to occur is that people may be afraid to be associated with the problem or emergency in a negative way. For instance, Cacioppo et al. (1986) found that people sometimes expect to be seen as the cause of someone’s misfortune. For example, the waiter would perhaps not help the choking man, because he or she fears that people may think the waiter in some way caused the coughing. Moreover, the waiter may be afraid to be sued if the help provided (e.g., Heimlich maneuver) was implemented incorrectly.

Thus, across a number of situations, the presence of bystanders exerts inhibitory effects on helping. Importantly, inhibition not only undermines helping, it also makes people respond more slowly—which is important, especially as in most emergency situations time matters greatly (e.g., Latané and Darley, 1969).

### Alcohol Consumption and Helping

Conventional wisdom holds that alcohol consumption is associated with multiple health and social concerns (Edwards, 1997). Despite the problems to individuals’ health, and the costly impact alcohol-related aggression and crime have on society, there could be some benefits associated with alcohol consumption: for example, alcohol can be used as a “social lubricant” which helps to smooth out social discourse (Monahan and Lannutti, 2000). Based on the notion that bystanders inhibit helping behavior, there are several ways in which alcohol consumption may help overcome the problem of bystanders intervening too slow or not at all. In the following section we will focus on the three most plausible processes according to the theoretical model outlined here.

#### Disinhibition

One of the main reasons alcohol changes social behavior is because it impairs cognitive functioning which is needed to inhibit certain social responses (e.g., intoxicated people are more likely to engage in self-disclosure, and sometimes aggression, than are non-intoxicated people (Steele and Southwick, 1985; Pernanen, 1991). This implies that the effects of alcohol may be very pronounced in situations in which people often inhibit their social responses, such as situations in which behaviors may yield negative consequences for one’s status, or which have potential physical risks (Steele et al., 1985). These are precisely the types of situations that arise during a bystander situation, and which lead people to question whether they should intervene or not. Indeed, a recent study showed that when people were asked to think about a situation in which they behaved without inhibition, they were quicker to respond and helped more overall in a subsequent task, even when there were bystanders present (van den Bos et al., 2009). Non-intoxicated (inhibited) people are capable of envisioning potential negative consequences of helping the choking man in the restaurant. For instance, they may fear embarrassment when it proves unnecessary. After some alcoholic drinks, however, cognitive functioning could already be impaired enough to not even consider embarrassment. In short, the anxiety of bystanders for embarrassment over misjudging a situation could be reduced, and people may not consider that they could be held responsible for the misfortune of the person in need.

#### Selective Attention

Alcohol myopia is related to the process of cognitive impairment. It entails that alcohol consumption makes people more attentive to salient cues in the environment, while it decreases attention to less salient cues (Steele and Josephs, 1990; Hirsh et al., 2011). This narrowing of one’s perception could lead to an increase in helping, because the behavior of the person in need, like a victim of an accident or crime is possibly more salient than the bystanders who do nothing. The person in need may get more attention than the bystanders, because he or she is the focal point of a situation which is often novel, uncommon (Berlyne and Ditkofsky, 1976), and sometimes dangerous (Grant and MacDonald, 2005). Not noticing the inaction of other bystanders could lead people to interpret the situation as more serious, and cause them to have less doubt about what to do (Latané and Rodin, 1969). Moreover, it could diminish the likelihood of bystanders to diffuse their sense of responsibility, because they may hardly notice the presence of other bystanders.

#### Behavioral Expectation

Alcohol consumption can even change bystander behavior without actually affecting their cognitive functioning. In general, people expect to behave less inhibited when they are drunk. The mere belief of people that they are intoxicated still yields uninhibited behaviors, even when people did not drink alcohol at all (Freeman et al., 2010). People sometimes use alcohol as an excuse for their behavior when they violate a social convention (Critchlow, 1986). This ‘excuse function’ of alcohol could make it easier for people to intervene, because when helping proves unnecessary or unwanted, they can simply blame alcohol for their behavior.

Based on a cost-benefit perspective of helping (Dovidio et al., 1991), consuming alcohol could lead people to help more and faster in the presence of others: people sometimes use helping as a means to obtain a good reputation, especially when they are aware that bystanders can see their prosocial behavior (Van Bommel et al., 2014). Moreover, after consuming alcohol, people sometimes become more prone to the benefits of social behaviors, and much less to the negative consequences. For instance, intoxicated people are more likely to engage in risky sexual behaviors (Davis et al., 2007). Without having to worry about the risk of intervening while others are present, the benefits may become clearer and thus, the decision to intervene becomes easier.

### Present Study

The present study was designed to provide an initial test of the notion that alcohol can diminish the negative influence of bystander presence on helping. In our pursuit of ecological validity, we faced several challenges. The most important challenge was that we wanted to provide a test in a real world, rather than the lab, as drinking in the lab may be quite a different experience from drinking in a more natural, social setting such as a bar. The study, therefore, takes place in actual bars in Amsterdam. We empirically test the notion that alcohol can diminish the negative influence of bystander presence on helping. We test this by measuring the proportion of helping and the onset time of helping during an item dropping paradigm in a bar (see also, Latane and Dabbs, 1975). During the item-dropping paradigm, we manipulate how many bystanders are present. Moreover, we measure the objective proportion of alcohol in participants’ blood by means of a breathalyzer.

In line with the bystander effect, we expect to find that the presence of bystanders decreases the amount of help given, and that it increases the time people need to come to a decision to help. However, our focus lies mostly on the interaction effect of alcohol and bystander presence. Alcohol is known to influence cognitive functioning (“The wine made me less reflective”; Hull, 1981; Hull et al., 1986) and perception (“I only saw the man choking”; Steele and Josephs, 1990), and alcohol can be used as an excuse (“The alcohol made me do it”; Critchlow, 1986). We therefore expect the presence of bystanders to have no effect when people have consumed alcohol. Alternatively, based on the notion that people who consumed alcohol may be more prone to see social benefits but not the risks, people who consumed alcohol are more likely and quicker to help, especially when others are around.

## Materials and Methods

### Participants and Ethical Statement

A total of 120 people (59 female, 61 male; mean age = 31.22, *SD* = 13.29) visiting one of four different middle-to-large sized bars in Amsterdam voluntarily participated, and were randomly assigned to the bystander present condition or the alone condition. We then measured their blood alcohol content with a breathalyzer (Alcovisor Sattelite, with a detection range from 0.000 to 0.400%). The BAC we measured ranged from 0.00 (completely sober) to 0.19% (roughly 8 standard units of alcohol in 1 h for a 160 pound adult male; 6 for a 140 pound female), *M* = 0.02, *SD* = 0.04. The ethical review board (VCWE) of the faculty of Psychology and Pedagogy of the VU University approved of this design. Upon entrance of the bar, thus prior to completing any questionnaire or being exposed to the item drop paradigm, participants signed an informed consent form. Participants could decide to withdraw their consent at any moment. At the end of the study participants were thoroughly debriefed and thanked for their participation. Participants were eligible to win a gift certificate worth €50,- (roughly $55,- in American currency).

### Procedure

Upon entering the bar, visitors were asked to sign an informed consent stating that they were willing to participate in a study later that evening. After at least 20 min, participants who agreed were asked to sit at a table at an isolated location inside the bar, where the experiment would take place. At this table, there were either two confederates (male) ostensibly filling out questionnaires, or there was no one at all. The two confederates sat next to each other, leaving two spots open on the other side of the table. The experimenter (female) sat next to the participant to explain what the questionnaire was about and that when the study was done, the participant would be asked to blow in the breathalyzer.

While the experimenter handed over the questionnaire, she deliberately knocked over a canister of 20 mouthpieces for the breathalyzer. The experimenter made sure every mouthpiece would fall out and roll over the floor. She would then hand over the questionnaire and slowly started picking up the mouthpieces at a rate of about one per 5 s, starting with the farthest first. At the time the canister was knocked over, an observer across the room, would start a stopwatch, and note down the time it takes the participant to engage in helping, and how long the participant would help. In the bystander condition, the bystanders were instructed to look up when the “accident” occurred, but they then continued filling out the questionnaire.

After the participants chose to help or not, and they finished the questionnaire, they were asked to blow in the breathalyzer. The BAC value was noted, the participant thanked and thoroughly debriefed.

### Measures

#### Helping Behavior

Helping behavior was measured two times. First, people were coded as helpers if picked up mouthpieces, or as non-helpers if they focused only on finishing their questionnaire. Second, helping behavior was quantified by counting the number of mouthpieces the participant picked up. This could range from 0 to 20 and was normally distributed (kurtosis = -0.46, skewness = -0.04).

#### Response Time

As soon as the experimenter dropped the jar of mouthpieces, a confederate to the experimenter in an adjacent room would start a timer on a stopwatch. The timer was stopped as soon as the participant picked up the first mouthpiece. There was no response time for people who did not help. Response times were log transformed to more closely fit normality assumptions (kurtosis = -0.38, skewness = 0.22 after transformation).

#### Alcohol Consumption

We measured drinking behavior in four ways: we measured BAC by means of a breathalyzer, but also asked how many alcoholic drinks they had during the last 3 h. Furthermore, we asked participants two questions, which they could answer on a seven-point Likert scale: “My current state is” with the answer anchors, 1 = *completely sober*, 7 = *heavily intoxicated*, and “At this moment alcohol influences my thought processes,” 1 = *completely disagree*, 7 = *completely agree*. These measurements were standardized using z-scores and combined into a reliable measure of alcohol consumption (α = 0.89). Finally, we log transformed this variable to more closely fit a normal distribution (kurtosis = 0.76, skewness = 0.69 after transformation).

## Results

### Helping Behavior

In line with research on the bystander effect we expected that the presence of bystanders diminishes helping. First, by the likelihood of people helping the experimenter or not, and second, by the number of mouthpieces picked up. We, however, also hypothesized that for people under the influence of alcohol, the bystander effect diminishes or even reverses.

First, we used binary logistic regression to test the effect of bystander presence (coded 0 for absent, 1 for present), alcohol consumption (centered) and their interaction term on the decision to help or not. The analysis showed that in the presence of others participants were (marginally) less likely to help (57.6% helped) than when they were alone (73.8%), Wald’s χ^2^(1, *N* = 120) = 3.479, *p* = 0.06. There was, however, no effect for alcohol consumption, nor an interaction effect (see **Table 1**.

**Table 1:** Helping behavior as a function of alcohol consumption and bystander presence.

	Response time.	Items picked up.	Helping yes/no.
	*B.*	*SE (B).*	.	*B.*	*SE (B).*	.	*B.*	*SE (B).*	Exp b.
Constant	0.51	0.42		11.38	0.54		-0.31	0.26	
Alcohol consumption	0.28	0.19	0.19	1.38	2.49	0.07	-0.42	1.46	16.74
Bystander presence	-0.02	0.06	-0.04	-3.19***	0.83	-0.41	-0.74	0.39	1.04
Alcohol consumption Bystander presence	-0.86*	0.33	-0.36	0.24	4.19	0.01	-1.92	2.15	10.01

*^∗^*p* < 0.05, ^∗∗∗^*p* < 0.001, ^†^*p* = 0.06. i: *R^2^* = 0.084, *F*(3,75) = 2.302, *p* = 0.084, ii: *R^2^* = 0.178, *F*(3,75) = 5.428, *p* = 0.002, iii: χ^2^ = 5.17, *p* = 0.16, R^2^ = 0.058 (Nagelkerke).*

Second, from those participants who actually helped (32 females, 47 males), we regressed the number of mouthpieces they picked up, on bystander presence (0 for not present, 1 for present), alcohol consumption (centered), and their interaction term. Consistent with the bystander effect, we found that the presence of bystanders diminishes the number of pieces participants picked up, *B* = -3.189, *t*(75) = -3.865, *p* < 0.001. However, there was no effect of alcohol consumption, nor an interaction effect (both *p*’s > 0.582), see **Table 1**

### Response Time

In line with research on the bystander effect, we expected that it would take people longer to engage in helping when others are present. However, we expected that alcohol consumption attenuates or reverses this bystander effect. We regressed reaction time (in seconds) on bystander presence (0 for not present, 1 for present), alcohol consumption (centered), and their interaction term (see **Table 1**.

**FIGURE 1:**
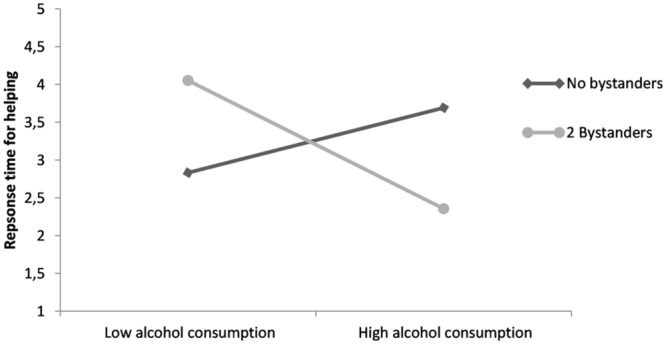
**Response time (untransformed) to engage in help as a function of bystander presence and alcohol consumption, plotted for 1 SD above and below average alcohol consumption**.

As expected, we found a significant interaction effect of bystander presence and alcohol consumption *B* = -8.55, *t*(75) = -2.618, *p* = 0.01.^1^ Simple slope analysis showed that relatively sober participants (-1 *SD* on the alcohol measure), responded 1.45 s slower when they were in the presence of bystanders than when they were alone, *F*(1,75) = 2.946, *p* = 0.09, albeit marginally. Conversely, people under the influence of alcohol (+1 *SD* on the alcohol measure), on average, responded 1.56 s faster in the presence of others than when they were alone, *F*(1,75) = 4.205, *p* = 0.04 (see also **Figure 1**.

## Discussion

Taken together, the results reveal that alcohol consumption indeed changes the classic bystander effect, in terms of reaction times. Alcohol consumption does not simply attenuate the influence of bystander presence on the decision making process, people who consumed alcohol actually became *faster* when others were around. People who consumed alcohol are sometimes slower to respond, as could be seen in the alone condition (**Figure 1**. This is in concurrence with previous findings (Maylor et al., 1987). In the presence of bystanders, however, participants who consumed alcohol responded much faster than sober participants. This provides support for the notion that alcohol makes people more action-oriented in the presence of others, as they are more readily prepared to take social risks. Helping can be used as a way of reputation management, especially when other people are around to witness the helpful behavior (Hardy and Van Vugt, 2006). Sober people, however, may focus too much on the potential risk of helping, while others are around, such as embarrassment or confusion of responsibility. For people under the influence of alcohol, however, the decision to help becomes much easier: they feel less inhibited and have the capability to shrug of potential social risk by blaming a possible negative outcome on alcohol.

In the introduction, we outlined three key processes that help understand how alcohol consumption could overcome the problem of bystanders intervening too slowly or not at all, namely disinhibition, selective attention, and behavioral expectations. In the current study, one of the first that examines the role of alcohol in bystander behavior, it was impossible to completely disentangle these potential explanations. However, the outcomes did help us to rule out some alternative hypotheses. For instance, based on the disinhibition hypotheses, one could have alternatively reasoned that alcohol lifts inhibitions toward selfish responses, such as not helping. Future research could aim to better disentangle the different processes we have outlined and perhaps even other, related, processes such as expectancies of self-confidence and heightened power after drinking. Two related processes which are shown to influence bystander intervention (e.g., Broeders, 2010). Obtaining more understanding about behavior of bystanders who consumed alcohol can potentially help increase the effectiveness of bystander intervention programs (such as described in Coker et al., 2011).

The foremost finding from the current contribution is that alcohol does not simply attenuate the entire bystander effect, but only increases *response speeds*. This could imply that the influences that seriously undermine helping and intervention (such as audience inhibition, confusion and diffusion of responsibility, and pluralistic ignorance) may slow down the decision process but do not actually change it. Indeed, people under the influence of alcohol in the presence of others were at least as fast as sober people who were alone. It seems that the complications caused by the presence of bystanders in the five-step model (Latané and Darley, 1970) may simply be ignored by them. Under the influence of alcohol, people may not notice that no-one is helping and thus do not experience pluralistic ignorance before step 2. They may not think that someone else will provide help and thus may not experience diffusion of responsibility before step 3, and they may not worry about how to implement the help in step 4. Because the amount of help given remains the same, it may imply that people make their decision to act rather quickly based on the immediate situation, and then contemplate and search (in each step) for information that confirms the correctness of the decision. As drunk people are often cognitively impaired, they may be less likely to go through this process of “false deliberation” (see also Waroquier et al., 2010).

We would like to note some strengths and limitations of the current work. Especially when making the step from the lab to the field, one must recognize the power of naturalistic influences. Small bars can be very noisy, both in a literal and methodological way. By inviting the participants to come to a secluded spot, we aimed to avoid such noises, but were not always completely successful. Another important limitation is that alcohol was only measured, not manipulated. Because of this limitation we must remain cautious about interpreting the results in terms of causality. At the same time, this is also adds to the major strength of the study, namely ecological validity. The experiment was conducted in a setting where the bystander effect may be very pronounced, and involved people who are often exposed to bystander situations, as bars are a common setting where people need help (e.g., accidents due to crowdedness, sickness due to alcohol intoxication, and public violence;(Allen et al., 2003). Taken together, these considerations suggest that in real-world public settings alcohol can have a substantial influence on the speed of helping, but also, that further study is necessary to more precisely establish the processes that operate here.

## Conclusion

The bystander effect has since the early studies (Latané and Darley, 1968) been described in terms of how likely people are to engage in helping behavior, and how quickly they help. The current contribution suggests that although these outcomes may be interrelated, they may be driven by different independent processes. Reflecting on the example of the choking man in the restaurant in the beginning of this paper, it may be clear that not many people will help the man when other bystanders are around, whether they just had some drinks or not. However, in the presence of others, the “drinker” may have come to action just in time, whereas the sober person may have elaborated so long that it was already too late. Indeed, the finding that alcohol can increase response time can hold very important outcomes, as when it comes to (the classic) bystander effect situations —such as crimes and sometimes horrible accidents— every second matters.

## Author Contributions

MvB and J-WvP designed the experiment, MvB Supervised data collection and prepared the first draft of the manuscript. All authors listed have made substantial, direct and intellectual contributions to the work by discussing the results and implications, and by commenting on and editing the manuscript at all stages.

## Conflict of Interest Statement

The authors declare that the research was conducted in the absence of any commercial or financial relationships that could be construed as a potential conflict of interest.

## Footnotes

1 Although not hypothesized, we repeated each key analysis with participants’ sex and the resulting interaction terms as additional variables, in order to test if the effects were different for males and females. We, however, found no significant interaction effect for sex. Helping behavior: Wald’s χ^2^(1, *N* = 120) = 0.133, *p* = 0.72; Number of mouthpieces: *B* = -0.490, *t*(71) = -0.462, *p* = 0.65; Response time: *B* = -0.301, *t*(71) = -0.378, *p* = 0.71.
